# Comparison of protein profiles of the pellicle, gingival crevicular fluid, and saliva: possible origin of pellicle proteins

**DOI:** 10.1186/s40659-020-0271-2

**Published:** 2020-01-15

**Authors:** Hibiki Odanaka, Takashi Obama, Naoko Sawada, Marika Sugano, Hiroyuki Itabe, Matsuo Yamamoto

**Affiliations:** 1 Department of Periodontology, Showa University School of Dentistry, Tokyo, Japan; 2 Division of Biological Chemistry, Department of Pharmaceutical Sciences, Showa University School of Pharmacy, Tokyo, Japan

**Keywords:** Pellicle, Saliva, Gingival crevicular fluid, Proteome, Isobaric tag labeling, Serotransferrin, Cystatin, Alpha-amylase

## Abstract

**Background:**

The pellicle, the acellular organic material deposited on the surface of tooth enamel, has been thought to be derived from saliva. In this study, protein compositions of the pellicle, gingival crevicular fluid, and saliva collected from healthy adults were compared to elucidate the origin of pellicle proteins.

**Results:**

The pellicle, gingival crevicular fluid, and saliva from the parotid gland or mixed gland were collected; subsequently, protein expression in samples from the respective individual was compared by SDS-PAGE and mass spectrometry. Following SDS-PAGE, proteins in the major bands were identified by mass spectrometry. The band pattern of pellicle proteins appeared different from those of gingival crevicular fluid, or saliva samples. Using mass spectrometry, 13 proteins in these samples were identified. The relative abundance of the proteins was quantitatively analyzed using mass spectrometry coupled with stable isotope labeling and by western blot. Cystatin S and α-amylase detected in pellicle were enriched in saliva samples, but not in gingival crevicular fluid, by western blot, and their abundance ratios were high in saliva and low in gingival crevicular fluid when analyzed by stable isotope labeling. Serotransferrin, however, was found only in the pellicle and gingival crevicular fluid by western blot and its abundance ratio was low in saliva.

**Conclusions:**

Our study revealed that the gingival crevicular fluid appears to contribute to pellicle formation in addition to saliva.

## Background

Pellicle is the organic material covering the tooth surface, but it does not contain bacterial materials. Pellicle is formed even within a few minutes after brushing and it is deposited on the surface of the enamel immediately after tooth eruption [1]. Formation of pellicle is a process by which oral proteins are selectively adsorbed onto the tooth enamel surface [2, 3]. Although it acts as an acid-resistant coating for teeth, pellicle also provides a foothold for the adhesion of bacteria [4], and its origin and function remain unclear.

Biochemical characteristics of pellicle were described in a previous review [5]. The ultrastructure of pellicle has been examined using electron microscopy previously and most studies report that the thickness of pellicle ranges from 30 to 100 nm, with granular structures having diameters of 25 to 125 nm [5]. The granules are thought to be pellicle proteins that bind the surface of hydroxyapatite through a stem-like structure. The amino acid composition of pellicle collected from variety of teeth was virtually the same, including the buccal side, the upper molar, upper jaw anterior teeth, and lower premolar teeth [6, 7]. The amino acid composition of pellicle and that of saliva were very similar; however, the pellicle formed in 2 h contained more hydrophobic amino acids but less proline than those in whole, mixed gland, and parotid saliva [5]. It has been suggested that salivary proteins are adsorbed onto the tooth surface preferentially over serum proteins. Serum proteins are able to attach to the tooth surface but they detach within a few hours after attaching [8]. When pellicle proteins recovered from different substrata were compared, pellicle protein deposited on a silica surface had a similar protein composition to that of pellicle deposited on natural teeth [9].

It is now feasible and practical to reliably identify a number of proteins from trace amounts of biological samples using liquid chromatography-tandem mass spectrometry (LC–MS/MS). To date, several proteome analyses have been carried out in order to determine the composition of pellicle proteins. Yao et al. analyzed pellicle proteins collected from healthy teeth and identified only four proteins: albumin, lysozyme, statherin, and cystatin [10]. Additionally, they performed a proteome analysis after separating proteins from pellicle and saliva by SDS-PAGE and reported differences in the protein composition [11]. A few studies showed pellicle proteins changed their composition during pellicle formation; some proteins including α-amylase, cystatin S, and proteins with calcium binding ability gradually decreased while increased were proteins involved in protein–protein interaction or neutrophil-derived proteins [12, 13]. Protein analysis of in vivo harvested pellicles collected from healthy adults 2 h after mechanical tooth surface cleaning by LC–MS/MS revealed that among 130 different proteins in pellicle 14% of them were derived from saliva, the majority (68%) originated from epithelial cells and the remaining (18%) from serum [14]. The process of pellicle formation could require selective adsorption and detachment of proteins at the enamel surface, however the origin of the proteins remains to be clarified.

Gingival crevicular fluid (GCF) is a plasma-derived exudate found in gingiva and grooves around teeth. GCF contains plasma proteins and inflammatory cells, and its production increases as periodontal disease progresses. Using isobaric tags for relative and absolute quantitation (iTRAQ) technique we previously analyzed relative quantitation of proteins in small amounts of oral samples and observed proteins from plasma and neutrophils are enriched in GCF [15]. A recent paper reported an increase in plasma protein was observed in the pellicle layer on the surface of the incisors of patients with gingivitis [16]. This observation led us to consider a possible involvement of GCF in pellicle formation. However, there have been no studies analyzing pellicle, GCF and saliva quantitatively from the same oral cavity simultaneously.

**Fig. 1:**
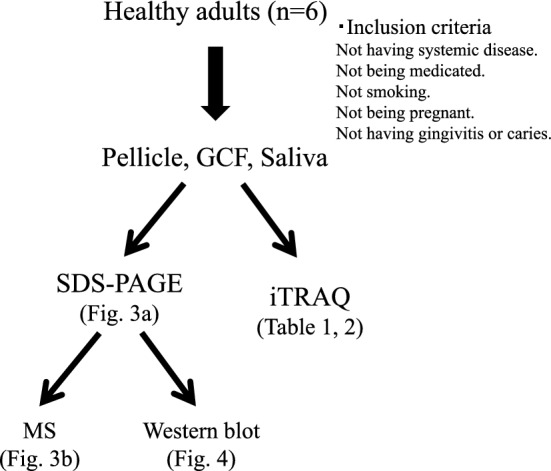
A schematic illustration of analyses performed in this study. Six healthy volunteers were recruited. A set of four samples (pellicle, GCF, two saliva samples from parotid and mixed glands) was collected from the same subject. These samples were analyzed by SDS-PAGE followed by MS and western blot and by iTRAQ quantitative comparison

In this study, a set of pellicle samples, GCF and saliva were taken from the same subjects. The protein profiles of the four types of samples were compared by SDS-PAGE, mass spectrometry, and quantitative analysis using iTRAQ technique to consider origin of pellicle proteins (Fig. 1).

## Methods

### Human subjects

Six healthy non-medicated volunteers, four males and two females, between 27 and 34 years old, were recruited. They had no overt sign of gingivitis or caries. Six sets of pellicles, GCF, and saliva samples were collected from the parotid gland and mixed gland (mixed saliva from submandibular gland and sublingual gland) of the same subjects, and the protocols for the sample collection were approved by the ethical committee of Showa University (No. 2016-011).

### Pellicle collection

After mechanical tooth surface cleaning, eating and drinking were prohibited for 2 h to allow pellicle formation. The tooth surface was washed twice with water and dried with air, and then 2/3rd of the crown side of the tooth surface was wiped with hydrophilic polyvinylidene fluoride (PVDF) membranes (Durapore membrane filter) immersed in 0.5 M sodium bicarbonate buffer (pH 9.0). Pellicle was collected from the upper and lower labial central incisor to buccal first molar [11]. The membrane was transferred into phosphate-buffered saline (PBS) containing protease inhibitor cocktail (Sigma-Aldrich, final 5% v/v) in 1.5 mL tube to elute proteins. Samples were frozen in liquid nitrogen and stored at − 80 °C until further use. After pellicle was collected, subsequently, GCF and saliva were collected.

### GCF collection

Before collection of GCF, the sampling sites were kept in isolation with cotton wool rolls, dried with air, and the tooth surface was wiped to prevent contamination by pellicle or saliva. Two paper points were inserted at three locations into the gingival sulcus of a single maxillary anterior tooth, after which the six paper points were placed in sterile PBS containing protease inhibitor [15, 17]. Samples were frozen in liquid nitrogen and stored at − 80 °C until further use.

### Saliva collection

After confirming the position of the parotid papilla or sublingual hills, the opening of each gland was wiped and saliva was collected by using two paper points. Since saliva from both submandibular and sublingual glands secret from the sublingual hills, this saliva is referred to mixed gland saliva. Saliva was absorbed onto the tip of a paper point for 10 s following which the paper point was cut and immediately placed in PBS containing protease inhibitor cocktail. Saliva collected using a total of eight paper points from a single subject was pooled in one tube. Samples were frozen in liquid nitrogen and stored at − 80 °C until further use.

### Identification of protein by SDS-PAGE

Protein concentrations in pellicle, GCF, and saliva samples were measured by Micro BCA Protein Assay reagents (Thermo, Rockford, IL, USA). To analyze protein patterns, gel electrophoresis was carried out on 15% polyacrylamide gel (ATTO E-T15S, ATTO Co., Tokyo, Japan) using EzRunT (ATTO AE-1415) buffer. After electrophoresis, the gel was fixed with 50% methanol and 10% acetic acid for 30 min and washed with distilled water three times, then soaked in flamingo staining solution for 1 h at room temperature. The image of the stained gel was recorded by Fluoro Phorestar 3000 (Anatech, Tokyo, Japan) with 10 s exposure. Each protein spot was punched out from the gel. The gel fragment was treated with 50 μL of 10 mM dithiothreitol/100 mM ammonium bicarbonate at 56 °C for 1 h. Then 50 μL of 55 mM iodoacetamide/100 mM sodium bicarbonate was added and the mixture was allowed to stand for 45 min with shading at room temperature. After washing with 100 μL of 50 μM ammonium bicarbonate/50% acetonitrile, the sample was dried thoroughly in a centrifugal concentrator VC-360 (TAITEC, Saitama, Japan). The sample was digested with 2 μL trypsin (100 μg/mL) overnight at 37 °C. Resulting peptides were extracted with 40 μL of 50% acetonitrile/0.1% trifluoroacetic acid for 30 min. After drying the extract, the sample was dissolved in 30 μL of 0.1% formic acid/2% acetonitrile. The LC–MS/MS analysis was performed as described previously [15].

### iTRAQ labeling

**Fig. 2:**
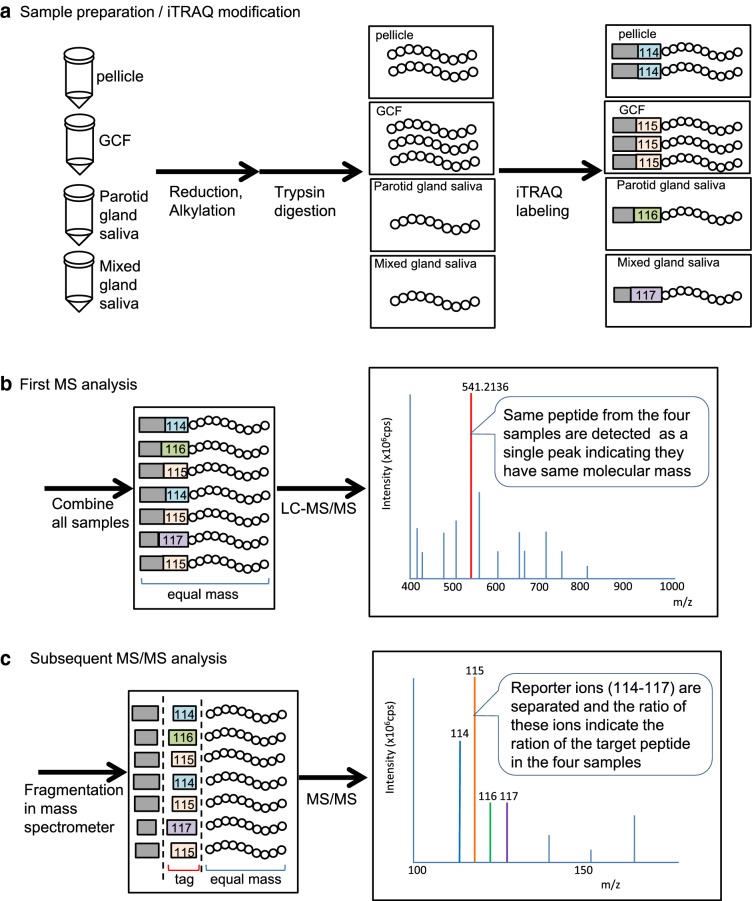
Schematic illustration of iTRAQ method of proteomic relative quantitation. **a** Pretreatment of the samples before LC–MS/MS analysis. A set of four samples from the same subject (5 μg each) was treated with dithiothreitol and iodoacetamide to cleave disulfide bonds followed by tryptic digestion. Then, amino termini of the peptides were labeled with either one of iTRAQ reagents 114, 115, 116 or 117. The iTARQ reagents have a same molecular mass but they produce isobaric tag moieties with different masses when cleaved during MS/MS analysis. **b** The iTRAQ-labeled four samples were mixed, and then the mixture was analyzed by LC–MS/MS. In the first MS analysis, the labeled peptides with the same molecular mass are detected as a single peptide. **c** The instrument subsequently breaks the peptides with high voltage and the fragments were analyzed. In this step, isobaric tags generated were detected separately. The ratio of the tags represents the ratio of the peptides in the four samples. In the same time, fragments of the peptides provide structural information about amino acid sequence

The procedure and basic principal of iTRAQ analysis is illustrated in Fig. 2. The same amounts of pellicles, GCF, and saliva samples containing 5 μg protein each were digested with trypsin and labeled with isobaric tags using iTRAQ multiplex kit (AB Sciex, Foster City, CA, USA). Trypsin solution (10 μL) was added to each sample tube, and the samples were incubated > 12 h at 37 °C. The four iTRAQ reagents containing isobaric tags with molecular mass 114, 115, 116 or 117 dissolved in 70 μL ethanol were transferred to the samples (114: pellicle, 115: GCF, 116: parotid gland, and 117: mixed gland) and incubated for 1 h at room temperature. The four iTRAQ-labeled samples were then combined in another tube. The organic solvent was removed, then the mixed sample was desalted and dried in a centrifugal concentrator. Finally, the sample was dissolved in 50 μL of 2% acetonitrile solution before subjecting to LC–MS/MS analysis. The LC–MS/MS analysis was performed as described previously [15].

### Western blot

For every sample, 5 μg quantities of proteins were subjected to SDS-PAGE per lane. Electro-transfer of the proteins to PVDF membrane was performed in a buffer (25 mM Tris–HCl/190 mM glycine/20% methanol/0.05% SDS) at 90 mA for 1 h. The PVDF membrane was recovered and immersed in blocking buffer containing skimmed milk. An anti-transferrin antibody (Abcam ab82411) and an anti-cystatin (Abcam ab151771) were diluted 1/1000 in 5% and 2% skimmed milk, respectively. An anti-salivary α-amylase antibody(ab201450)were diluted 1/2000 in 2% skimmed milk. The membrane was immersed and shaken for 2 h at 25 °C. Horseradish peroxidase-conjugated anti-rabbit IgG antibody was added to the membrane as a secondary antibody for 2 h. The protein surface of the PVDF membrane was covered with a chemiluminescence reagent (ECL primer) for 5 min; then data were recorded in a chemiluminescence detection apparatus (LAS500).

### Statistical analysis

Statistical significance of the protein abundance was calculated from the data obtained by iTRAQ analysis using Kruskal–Wallis test. P-values less than 0.05 were considered as significant.

## Results

**Fig. 3:**
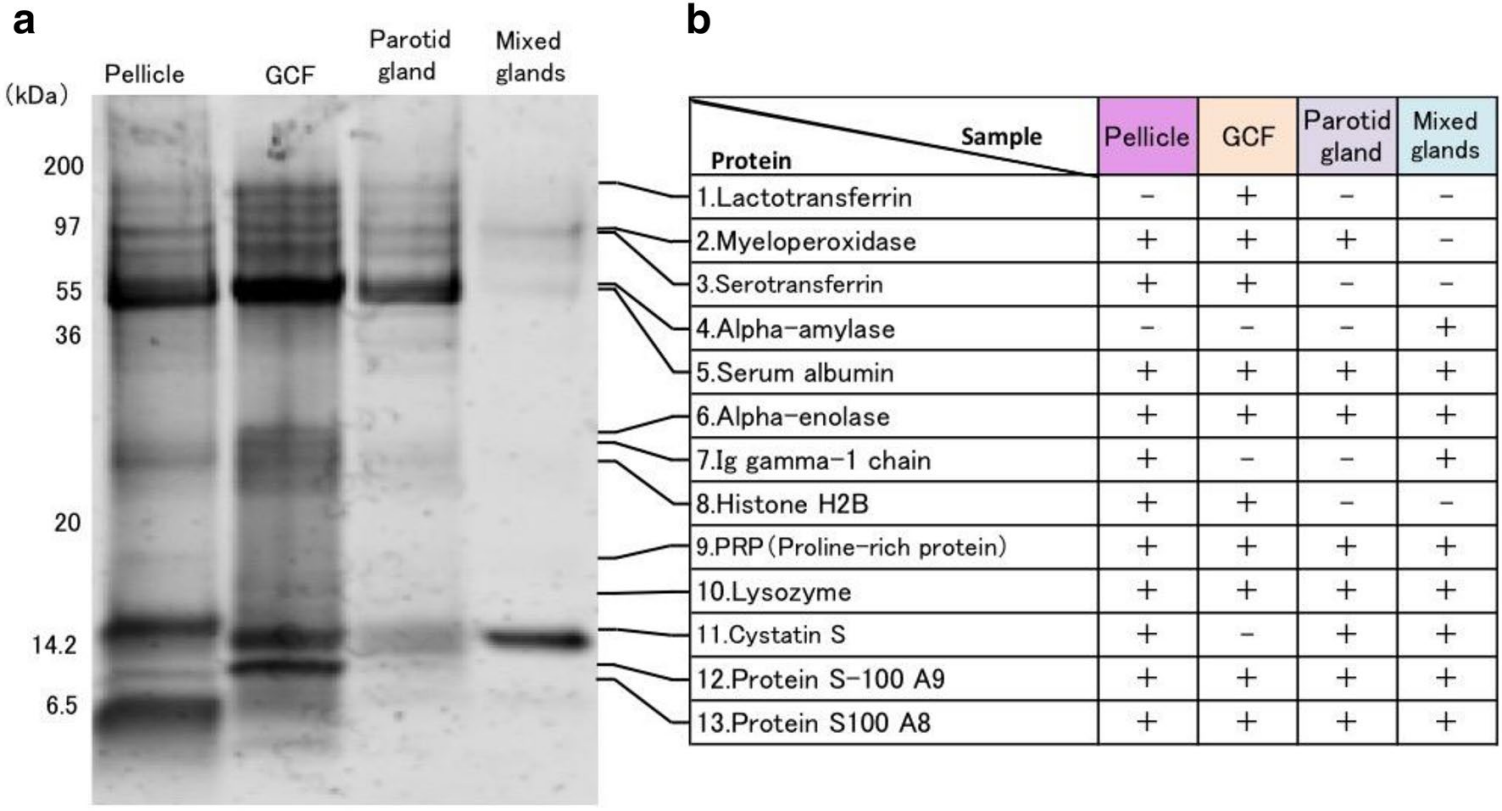
SDS-PAGE and protein identification of each sample. **a** Samples (5 μg/lane) from pellicle, GCF, parotid gland, and mixed gland, were analyzed on SDS-PAGE (15% acrylamide). **b** Proteins in the major bands(1–13)were identified by mass spectrometry. The bands from the gel were punched out and analyzed by LC–MS/MS. Pellicle, GCF, parotid saliva, and mixed gland saliva contained 11, 10, 9, and 9 of the 13 proteins identified, respectively

A set of four samples, pellicle, GCF, saliva from parotid grand, and saliva from mixed gland, was collected from each healthy volunteer. Proteins in all the samples were separated by SDS-PAGE and identified by MS. The band patterns of proteins were visualized by flamingo staining after electrophoresis (Fig. 3a). In each sample, several characteristic bands were observed. The major bands were punched out from the gel, and the compositions of the proteins contained in these bands were analyzed by LC–MS/MS. The proteins found in each band were identified based on the peptide fragment data with high reliability. The pellicle collected in this study was formed within 2 h after mechanical tooth surface cleaning and 13 kinds of proteins were identified in the newly formed pellicle (Fig. 3b). Three dense protein bands were observed at 55, 16, and 6 kDa. The band at 55 kDa was identified as serum albumin (band 5) and was found in all samples. Cystatin S was identified at 16 kDa bands (band 11), and it was detected in pellicle and saliva samples by MS. A characteristic band was observed in the pellicle at 6 kDa. Based on the molecular weight data the band is likely to be statherin; however, this could not be confirmed by analyzing the data from MS. Among the 13 proteins identified, some were found in all samples, while some were found only in pellicle and GCF (e.g., serotransferrin, band 3) and others were found only in pellicle and saliva (e.g., cystatin S, band 11).

Furthermore, the set of four samples was mixed, and LC–MS/MS was performed by the iTRAQ procedure (Fig. 2). Table 1 lists the top 50 proteins identified in a mixed sample of pellicle, GCF, and saliva from parotid and mixed glands are shown. A number of proteins were identified by the MS analysis, and it is assumed that proteins with a score ≥ 20 are reliable. All of the major proteins identified after SDS-PAGE (Fig. 3b) appeared among the top 50 proteins listed in Table 1 except for histone H2B.

**Table 1:** Proteins identified in pellicle, GCF, and saliva samples

No	Accession number	Score	%Cov	Name	Peptides (95%)
1	P02768	132.13	85.1	Serum albumin	160
2	P19013	124.83	88	Keratin, type II cytoskeletal 4	148
3	P13646	118.77	78.6	Keratin, type I cytoskeletal 13	186
4	P02538	112.25	71.8	Keratin, type II cytoskeletal 6A	118
5	P02788	93.78	76.3	Lactotransferrin	65
6	P01833	84.02	59.4	Polymeric immunoglobulin receptor	90
7	P04745	79.37	81.2	Alpha-amylase 1	129
8	P15924	77.69	33.2	Desmoplakin	44
9	P02533	81.99	83.7	Keratin, type I cytoskeletal 14	75
10	P02812	67.59	88.9	Basic salivary proline-rich protein 2	
11	P01876	64.9	91.2	Ig alpha-1 chain C region	101
12	P13647	99.44	70.5	Keratin, type II cytoskeletal 5	88
13	P02787	56.01	58.6	Serotransferrin	31
14	P04083	46.39	75.7	Annexin A1	36
15	Q9HC84	45.57	10.1	Mucin-5B	23
16	O60437	45.31	28.6	Periplakin	25
17	P01036	41.83	72.3	Cystatin-S	119
18	P08779	80.32	79.1	Keratin, type I cytoskeletal 16	67
19	P63261	37.63	80.3	Actin, cytoplasmic 2	33
20	Q9UBC9	37.18	93.5	Small proline-rich protein 3	20
21	P01871	36.8	59.5	Ig mu chain C region	26
22	P25311	35.97	67.1	Zinc-alpha-2-glycoprotein	26
23	P04280	66.34	88.5	Basic salivary proline-rich protein 1	62
24	P05164	34.63	48.5	Myeloperoxidase	23
25	P02810	34.35	72.3	Salivary acidic proline-rich phosphoprotein	77
26	P13645	53.41	63.2	Keratin, type I cytoskeletal 10	42
27	P06733	34	69.8	Alpha-enolase	23
28	P14618	33.39	52	Pyruvate kinase isozymes M1/M2	19
29	P31947	31.24	76.6	14-3-3 protein sigma	19
30	P22079	30.42	43	Lactoperoxidase	17
31	Q09666	29.74	24.3	Neuroblast differentiation-associated protein	21
32	P07355	29.14	54	Annexin A2	17
33	P61626	28.07	63.5	Lysozyme C	47
34	Q9UBG3	26.93	54.1	Cornulin	25
35	Q9UGM3	26.74	37.9	Deleted in malignant brain tumors 1 protein	16
36	P01857	26.64	79.1	Ig gamma-1 chain C region	29
37	P05109	26	90.3	Protein S100-A8	23
38	Q92817	25.4	21.5	Envoplakin	13
39	P12273	24.91	78.1	Prolactin-inducible protein	30
40	Q01546	41.09	51.7	Keratin, type II cytoskeletal 2	31
41	P01024	23.14	19.4	Complement C3	18
42	A8K2U0	22.13	22.3	Alpha-2-macroglobulin-like protein 1	14
43	Q08188	21.57	38.1	Protein-glutamine gamma-glutamyltransferase E	12
44	P04406	21.56	58.5	Glyceraldehyde-3-phosphate dehydrogenase	15
45	P06702	21.49	91.2	Protein S100-A9	38
46	P14923	19.71	34.8	Junction plakoglobin	14
47	Q8N1N4	31.48	43.1	Keratin, type II cytoskeletal 78	20
48	P08107	19.12	19.5	Heat shock 70 kDa protein 1A/1B	10
49	P09228	31.05	76.6	Cystatin-SA OS = Homo sapiens	73
50	P07476	18.23	32.5	Involucrin OS = Homo sapiens	12

The top 50 proteins identified in the mixed sample of pellicles, GCF, parotid saliva, and mixed saliva are listed. During iTRAQ analysis, a mixture of the four samples was analyzed by LC–MS/MS quantitatively. All of the major proteins in Fig. 3b except for histone H2B are among the top 50 proteins in Table 1. Note that keratins may be originated from surrounding tissue or environment. N: number of identified proteins. The proteins are listed in the order of reliability evaluated by the “score” and the “%Cov”. Score: A value indicating the reliability of the identification calculated from the accuracy of the amino acid sequence of the fragments, the intensity of peptide signals, coverage of the whole protein sequence, and number of fragments detected.  % Cov: The ratio of amino acid number identified from the peptides to the whole sequence of the protein. Peptides (95%): The number of peptide fragments reliably identified for the protein

Relative abundance of the 13 pellicle proteins in the GCF and saliva samples was quantitatively evaluated for the six sets of samples collected from six subjects using the iTRAQ proteomic procedure (Table 2, Fig. 2). Amount of α-amylase was constantly lower in the GCF than in the pellicle and higher in the saliva samples from the parotid and mixed glands than in the pellicle sample in all six subjects. There was a significant difference between GCF and mixed gland saliva. The amount of cystatin S was significantly lower in GCF than in pellicle and higher in saliva than in pellicle. Serotransferrin was constantly higher in GCF than in pellicle samples in all six subjects and lower in saliva samples from parotid and mixed glands. Amount of myeloperoxidase (MPO) tended to be lower in saliva samples from parotid and mixed glands than pellicle, and serum albumin, α-enolase and Ig gamma-1 chain levels were higher in GCF samples, although they were not statistically significant.

**Table 2:**
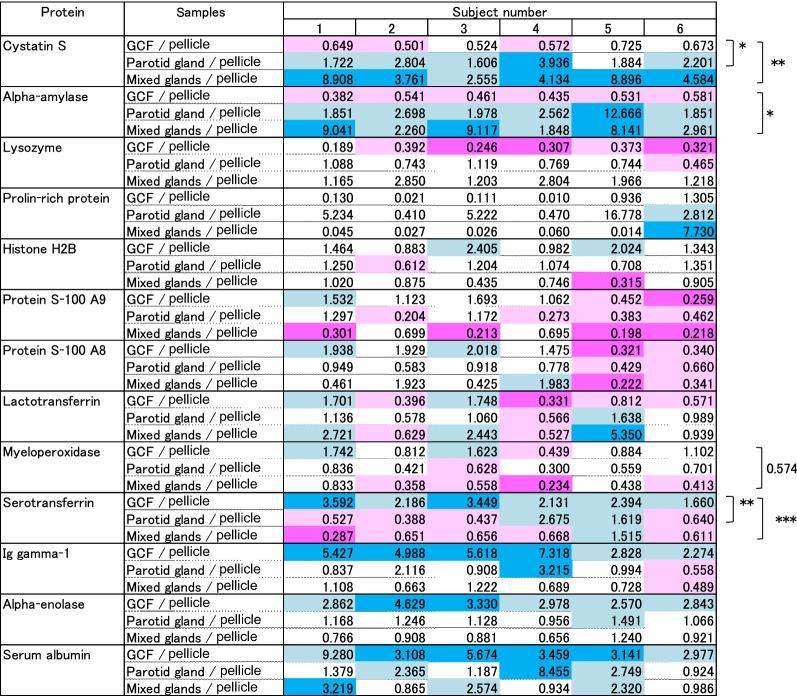
Relative ratios of the major proteins in pellicle quantified by iTRAQ

Quantitative analysis of the proteins in the pellicle, GCF, saliva from parotid gland and saliva from mixed gland was performed using iTRAQ labeling. Amount of each protein in pellicle was compared with those in GCF or two saliva samples among each subject (n = 6). The ratio calculated for each protein was indicated in the table; colored tiles represent ratio ≥ 3 (blue), ≥ 1.5 (sky), ≤ 0.67 (pink) or ≤ 0.33 (magenta), respectively. When the data was not reliable enough based on consistency of peptide signals in MS/MS the tile was left uncolored. Statistical analysis was carried out by Kruscal–Wallis test. P values ≤ 0.05, *; P ≤ 0.01, **; P ≤ 0.005, ***

**Fig. 4:**
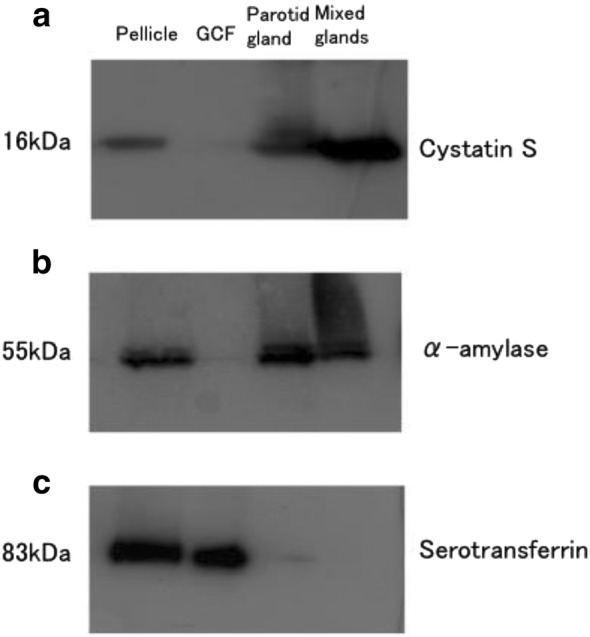
Detection of pellicle proteins by western blot. Samples (5 μg/lane) from pellicle, GCF, parotid gland, and mixed gland, were subjected to SDS-PAGE. Three pellicle proteins, cystatin S (**a**), α-amylase (**b**), and serotransferrin (**c**), were examined by western blot

To confirm the differences in distribution of these three proteins, western blot analysis was carried out (Fig. 4). Salivary α-amylase and cystatin S that are known to be present in pellicle were detected in the pellicle, parotid gland, and the mixed gland, but not in GCF. Serotransferrin was detected in pellicle and GCF but not in saliva samples.

## Discussion

This study aimed to quantify the relative amounts of proteins in pellicle, GCF, and saliva collected from the oral cavity of healthy subjects. It has been generally thought that pellicle is derived from saliva, but we have demonstrated that both saliva- and GCF-derived proteins are included in the pellicle.

Having identified many proteins with different abundance levels in the oral samples of healthy subjects by SDS-PAGE, we carried out MS using the iTRAQ method to compare relative protein abundance of the identified proteins quantitatively, confirming that levels of proteins in pellicle, GCF and saliva differ in these subjects. Cystatin S was not detected in GCF either by SDS-PAGE or western blot, and it was also low in abundance according to iTRAQ analysis. α-Amylase was present in pellicle and saliva but not in GCF (by western blot) and was low in abundance in GCF (by iTRAQ). These results strongly suggest that α-amylase and cystatin S in pellicle are derived from saliva.

Serotransferrin was detectable in the pellicle and GCF samples but barely detectable in the saliva samples by western blotting. Relative quantitation by iTRAQ showed significantly lower abundance of serotransferrin in saliva than pellicle. Overall, these results suggest that serotransferrin is derived at least in part from GCF. Given that the serum protein concentration is 70 times higher in GCF than that in saliva [8], we consider that some of the GCF-derived proteins may be contributing to pellicle formation. Serotransferrin, also called plasma transferrin, is a ferrous ion binding protein that functions to deliver iron to cells and to provide anti-bacterial, low-iron environment [18]. It is possible to assume serotransferrin could have an antibacterial role at the tooth surface.

Cystatin is a group of proteins that exhibit cysteine protease inhibitory activity. Several secretory cystatins, including cystatin S, have been separated and characterized previously [19]. Peptide fragments derived from cystatin S inhibit the proliferation of the periodontal pathogen *Porphyromonas gingivalis* [20]. Cystatins act not only on bacteria but also on host cells to induce cytokine production, and they are also involved in acquired immune responses. Our results confirm that, among pellicle proteins, cystatin S is derived from saliva as reported previously. Salivary α-amylase is secreted from the parotid gland in the oral cavity and is a protein specific to saliva. This enzyme irregularly cleaves the α-1,4-bond of starch or glycogen producing maltose or oligosaccharide. It is well known that salivary amylase activity is highly correlated with the plasma norepinephrine concentration and consequently is used as an indicator of stress in the sympathetic nervous system [21]. α-Amylase contributes to dental plaque formation, since amylase-binding-protein A interacts with salivary amylase and inhibits the formation of dental plaque caused by *Streptococcus gordonii*, an early colony-forming bacterium [22].

SDS-PAGE analysis showed a characteristic band at 6 kDa in the pellicle gel pattern (Fig. 3a). This is likely to be statherin, which is an acidic protein specifically found in pellicle [3, 4, 12]. Even though we repeatedly attempted to identify this protein by MS, we were unsuccessful probably because the statherin sequence contained few arginine and lysine residues making it very difficult to obtain tryptic fragments suitable for MS analysis.

Some of the pellicle proteins seems to relate to local inflammation and may be derived from neutrophils which have an important antimicrobial role in the innate immune system. MPO is the most abundant protein in neutrophils and monocytes. Opsonized bacteria are captured by these phagocytic cells; MPO and other antimicrobial systems in cytoplasmic granules fuse and release into the phagosomes to kill them [23]. Protein S-100 A8 and A9 are calcium binding proteins that form a heterodimer called calprotectin. Calprotectin is a major cytoplasmic protein in neutrophils and has an antibacterial role by removing manganese and iron [24].

Saliva acidic PRP, the main component of saliva, binds to hydroxyapatite via negatively charged amino acid residues and is partly linked to the pellicle. PRP was detected in our experiments, but the abundance was not consistent. It is reported that human leukocyte elastase had ability to remove the acidic PRP’s negatively charged N-terminal part in saliva, suggesting an interfering effect of elastase on PRP deposition. However, when acidic PRP is bound to hydroxyapatite, elastase cannot digest it [25].

In this study, quantitative analysis was carried out to obtained protein profiles of the same amount of pellicle, GCF and saliva samples, however the ratio of their contributions to pellicle proteins cannot be estimated from our study. There must be additional factors to be considered that affect pellicle formation such as total volume of GCF and saliva, accessibility of those fluids to tooth surface.

## Conclusions

Our study demonstrates that pellicle contains proteins derived not only from saliva but also from GCF. Thus, in addition to its roles in periodontal tissues reported to date, GCF could have a role in pellicle formation on the tooth surface. Further research is recommended to elucidate the role of GCF in pellicle formation and function.

## Data Availability

The datasets used and/or analyzed during the current study are available from the corresponding author on reasonable request.

## Abbreviations

GCF: gingival crevicular fluid
ICAT: isotope-coded affinity tag
iTRAQ: isobaric tag for relative and absolute quantitation,
LC–MS/MS: liquid chromatography–tandem mass spectrometry
MPO: myeloperoxidase
MS: mass spectrometry
PBS: phosphate-buffered saline
PRP: proline-rich protein
PVDF: polyvinylidene fluoride
SDS-PAGE: sodium dodecyl sulfate-polyacrylamide gel electrophoresis
